# Comparing the Diagnostic Outcomes of Staining Various Breast Lesions with Either Anti-CK 5/6 or Anti-CK 5

**DOI:** 10.30699/IJP.14.2.113

**Published:** 2019-06-10

**Authors:** Mihir A Gudi, Chung Y Yi, Mahrokh Nohadani

**Affiliations:** 1 *DPLM, KK Women’s and Children Hospital, Singapore*; 2 *MSc, Department of Surgery and Cancer, Hammersmith Hospital, Imperial College London, England, UK*; 3 *Department of Histopathology, Hammersmith Hospital, Imperial College London, England, UK*

**Keywords:** Immunohistochemistry, Disease of Breast, Monoclonal Antibody

## Abstract

**Background and Objective:**

Anti-CK5/6 monoclonal antibodies have an established role in breast disease diagnosis. Anti-CK5 monoclonal antibodies have recently become commercially available. There has been growing interest in the staining characteristics of anti-CK5 and its potential diagnostic role in place of anti-CK5/6. We aim to compare and contrast the staining characteristics of anti-CK5/6 vs anti-CK5.

**Material and Methods:**

58 tissue blocks containing 122 different lesions were selected from tissue archives. Two specimens (groups) were taken from each lesion One (group) was stained with anti-CK5 and the other (group) with anti-CK5/6 monoclonal antibodies, using the Streptavidin-biotin immuno-peroxidase method. The two groups of slides were compared and contrasted for lesion staining pattern and for intensity, using light microscopy.

**Results:**

Results showed that the diagnostic staining pattern was exactly the same in both anti-CK5 and anti-CK5/6 groups, and also showed that anti-CK5, stained most of the lesions more intensely than anti-CK5/6.

**Conclusion:**

Anti-CK5 performed at least as well (for lesion-pattern staining), and better (for lesion staining intensity) than did anti-CK5/6 in the diagnosis of a wide range of breast tissues and lesions. It may be justified to safely replace anti-CK5/6 with anti-CK5 in future routine clinical use, with resultant diagnostic and economic benefits.

## Introduction

The increasing incentive to pick up breast lesions at an earlier pre-malignant stage, for example by mammography of asymptomatic patients, has successfully enabled extremely small non-palpable tumors to be detected (1,2). Theses premalignant/malignant lesions are like: atypical ductal hyperplasia (ADH), lobular carcinoma in situ (LCIS), ductal carcinoma in situ (DCIS), and invasive carcinoma (3). The lesions share certain histologic features irrespectively. It is often difficult to clearly distinguish one lesion from another differential diagnosis, especially as they are often seen together in proximity and often intermixed in the same tissue sample. There is also some overlap between the official criteria used to diagnose some of these atypical and premalignant lesions, which adds to the confusion.

There are at least three problematic and commonly seen areas where the standard haematoxylin and eosin (*H&E*) staining alone, may be inadequate in diagnostic breast histopathology assessment:

One such problem area is in distinguishing between benign papilloma vs papilloma with atypia, and between papilloma vs intracystic papillary carcinoma, and also between papilloma vs papilloma with DCIS.

These lesions may look similar and could pose diagnostic difficulties in differentiating from one another on *H&E* sections alone. 

Another common problem area is in distinguishing between usual ductal hyperplasia (UDH) vs atypical ductal hyperplasia (ADH), and also between UDH vs low-grade ductal carcinoma in situ (DCIS). UDH, ADH and low grade DCIS, may have overlapping features between themselves under the light microscope when stained with the *H&E*, and may be difficult to distinguish apart reliably. Distinguishing between the lesions has important clinical implications. UDH is considered a benign lesion with very little potential risk of associated carcinoma, and no treatment is indicated, whereas DCIS and LCIS are considered higher risks of subsequent and associated invasive carcinoma and treatment is indicated. 

Another common problem area is in distinguishing between benign or premalignant, but non-invasive lesions such as radial scar and sclerosing adenosis and DCIS vs invasive cancer. 

For many years, histopathologists have only had one additional diagnostic stain to assist with the above difficult diagnostic situations, the Dako (DakoCytomation) anti-CK5/6 monoclonal antibody besides ER (9). These anti-CK 5/6 antibodies, recognize both CK5 and CK6 (and also CK4) in breast lesions. The relative contribution of the anti-CK5 and the anti-CK6 activities of the Dako monoclonal antibody in the diagnosis of breast disease has not yet been elucidated. It is unknown whether the staining seen with anti-CK5/6 monoclonal antibody is due mainly or in part to the anti-CK5 or the anti-CK6 activity (or whether both have equal importance in its use). Indeed, details concerning the relative expression of the antigens CK5 and CK6 on various breast lesions as yet remain unknown. There is some suggestion that with the anti-CK5/6 monoclonal antibody, the contribution of the anti-CK6 activity in breast cancer diagnosis is possibly less important than the anti-CK5 activity (10-15). However, Leica Microsytems has recently produced and made a monoclonal antibody specific to CK5 alone which is commercially available.^16^ This has allowed further studies into the understanding of the relative expression of CK5 and CK6, and their importance in the immunohistochemistry of various breast lesions, and has merited the following investigation.

In the current era where cost has a large bearing on the investigations requested in the diagnostic setting, having CK5 vs CK5/6 as an alternative antibody may lead to the cost reduction and hence would be beneficial. 

## Materials and Methods

**Case selection**

Using the Imperial College Healthcare Trust pathology data retrieval *CoPath* computer system, a selection of 267 patients diagnosed with any breast disease from 10/11/2009 till 8/11/2010, who had either a WLE or a mastectomy (i.e. patients who had core biopsies were excluded) were selected. The final pathology report for each case was extracted from the *CoPath* computer system. All the diagnoses of breast disease on the report and their corresponding block number/s were also noted and listed. The corresponding *H&E* slides of the identified blocks, were extracted from the slide archives. These slides were all `re-read` under the light microscope. This step was deemed necessary to personally re-confirm the diagnoses, and all the lesions seen were documented. A selection of 58 of the highest yield slides was made from the above re-read slides, which included approximately 10-14 of each of a range of lesions, ranging from normal tissue, benign lesions, premalignant lesions to invasive malignant lesions. A decision was made at this stage to have as many lesions per slide as possible to maximize lesion number for the study, due to limitations of cost and manpower of having just one lesion per patient, i.e. choosing slides with just one lesion. The wax paraffin tissue blocks corresponding to the cases/slides were then extracted from the wax paraffin specimens and then stained.

**Laboratory procedures and protocols**

The experiments were carried out in two separate `runs`. Two sections (three in case of the control experiments) were cut from each block and each section was mounted onto a microscope slide. Test experiments were performed to ascertain the optimal antibody dilutions for the main experiments. An optimal dilution of 1:150 was ascertained for both the anti-CK5/6 and anti-CK5 antibodies, and this dilution was used to stain both the anti-CK5/6 and anti-CK5 slide populations. The sections were then processed and stained in two separate `runs`, rather than in just single `run`, and this was due to the real limitations of manpower and time available (The corresponding anti-CK5/6 and the anti-CK5 slides of each block/case were always done in the same `run`). One control experiment was performed per `run`. The control experiment would consist of three sections of a lesion known to stain positively to anti-CK5/6. One section would be untreated with either antibody (negative control) and one (positive control) would be treated with anti-CK5/6 and one (other positive control) with anti-CK5. The sections were then processed as per protocol described by Polak & Van Noorden.^17^

**Interpretation of the stained slides**

All slides were analyzed using an Olympus BX51 microscope (Olympus, Essex, UK), under x20, x40, x100 and x200 magnifications. Before analyzing any test slide, the positive and negative control slides for that particular `run`, were looked at, to ensure appropriate positive staining in the positive controls and appropriate negative staining in the negative controls, and so assure the validity of the staining results of the other/remaining study slides. The *H&E* slide corresponding to a particular pair of anti-CK5/6 and anti-CK5 slides, was first examined, before viewing the test slides, to replicate as far as possible the real clinical diagnostic sequence in this study. No commitment to any diagnosis (or diagnoses) was made after viewing the *H&E* slide. The corresponding anti-CK5 and anti-CK5/6 stained slides were then analyzed in tandem (placed side by side under the light microscope). The decision to analyze them in tandem was to allow for better comparison and contrasting of the staining intensities and also the lesions diagnosed in the two differently stained slides. The investigator (a consultant breast histopathologist) viewing the slides was blinded to both the previously reported `gold standard ` diagnoses previously made and listed, and to the identity of the stain of the slides, given in tandem, (ie whether anti-CK5/6 or anti-CK5), reported all lesions separately seen in each slide. The investigator then reported the staining intensity of each lesion seen in both slides, using a scoring scale of: *Score 0= no staining, Score 1= weak staining, Score 2= moderate/average staining, Score 3= intense staining.* This scoring system used was devised by the investigators and was based on the `score for intensity` of the quick (Allred) score system used for assessing steroid receptor staining characteristics in breast cancer diagnosis.^18^ The designation of a particular score/grade (0-3) to each lesion was based on the experience with interpreting anti-CK5/6 stained slides, and also upon experience with use of the scoring system in the quick (Allred) score system^19^, and also finally based on the availability in this study to directly compare the anti-CK5/6 and the anti-CK5 slides placed in tandem side by side under the light microscope. After all the slides were analyzed for diagnosis and staining intensity, the authors then compared the diagnoses made of the anti-CK5/6 and the anti-CK5 stained lesions in the study with the `gold standard` diagnoses (made and listed earlier) of the lesions chosen for the study.

**Photographic recording of lesions**

Photographs of selected classic examples of a range of stained lesions were taken using an Olympus BX51 microscope (Olympus, Essex, UK) with an attached Olympus C-5060 digital camera body (Olympus, Essex, UK). 

**Statistical methods**

The numerical data were tabulated and the McNemar test for categorical data (using SPSS software) was used to compare the results of individual lesions staining with either anti-CK5/6 or anti-CK5. One-sided exact *P-value* results were used to see whether staining with anti-CK5 resulted in an improved staining intensity. The McNemar test (one-sided) directly compares whether an individual`s particular lesion when stained with anti-CK5/6, will stain more intensely or not when stained with anti-CK5 (a one-tailed test will be used to test for improvement in the staining only). We were only looking for any improvement in staining from an anti-CK5/6 stained lesion scoring 0 or 1 or 2, to a score of 3 if stained with anti-CK5. For the purpose of simplifying this test, we did not look at any improvement in scores from 0 to 1 or from 1 to 2. 

**Ethics approval**

The application was approved by the Imperial College Trust`s Tissue Management Committee. 

## Results

**Control slides**

Both the positive and negative controls were appropriately stained and non-stained, respectively. They were confirmed as such; interpreted at the beginning of the two slides interpretation meetings by the investigators, and before the slides from a particular staining `run` (a total of 2 `runs`). 

**Staining patterns (lesion diagnosis)**

The total number of different lesions diagnosed /identified in the anti-CK5/6 and the anti-CK5 stained slide populations are summarized below (Table 1).

**Staining intensity**

The distribution of the staining intensity scores of all the anti-CK 5/6 stained lesions vs all the anti-CK 5 stained lesions are shown in Table 2. 

 The distribution of the staining intensity scores of the different individual lesions stained with anti-CK 5/6 vs staining with anti-CK 5 are shown in Table 3.

The results of the statistical analysis (McNemar Tests) are shown in Table 4.

**Table 1:** Comparison of the different lesions distribution diagnosed in both anti-CK 5/6 and anti-CK 5  stained sample population

Lesions seen on the slide (diagnoses made)	Total number in anti-CK 5/6 group	Total number in anti-CK 5 group
**Normal tissue**	10	10
**FCC/AM**	16	16
**SA**	12	12
**CCC**	14	14
**FA**	12	12
**Papilloma**	14	14
**Hyperplasia/UDH**	12	12
**ADH/DCIS**	18	18
**Invasive carcinoma**	14	14
**Other**	0	0
**Total**	122	122

**Table 2:** Comparison of the distribution of staining intensity scores of the two groups of slides stained with either anti-CK5/6 or with anti-CK5

Staining intensity score of the lesions seen on the slide (Score range = 0-3)	Total number in anti-CK 5/6 group	Total number in anti-CK 5 group
**0 (no staining)**	14	14
**1 (weakly staining)**	3	0
**2 (moderately staining)**	69	4
**3 (intensely staining)**	36	104
**Total**	122	122

**Table 3:** Comparison of the staining intensity scores of all the individual lesions for the anti-CK 5/6 and the anti-CK 5 populations

Lesion	Anti- CK 5/6 (freq) staining grade	Anti- CK 5/6 (%) staining grade	Anti- CK 5 (freq) staining grade	Anti- CK 5 (%) staining grade	Total number of each lesion
Normal tissue	Grade 0=0 Grade 1=0 Grade 2=6 Grade 3=4	= 0% = 0% = 60% = 40%	Grade 0=0 Grade 1=0 Grade 2=0 Grade3=10	= 0% = 0% = 0% = 100%	10
FCC/AM	Grade 0=0 Grade 1=0 Grade2=11 Grade 3=5	= 0% = 0% = 69% = 31%	Grade 0=0 Grade 1=0 Grade 2=0 Grade3=16	= 0% = 0% = 0% = 100%	16
SA	Grade 0=0 Grade 1=0 Grade 2=9 Grade 3=3	= 0% = 0% = 75% = 25%	Grade 0=0 Grade 1=0 Grade 2=0 Grade 3=12	= 0% = 0% = 0% = 100%	12
CCC	Grade 0=0 Grade 1=0 Grade2=11 Grade 3=3	= 0% = 0% = 79% = 21%	Grade 0=0 Grade 1=0 Grade 2=1 Grade3=13	= 0% = 0% = 7% = 93%	14
FA	Grade 0=0 Grade 1=2 Grade 2=6 Grade 3=4	= 0% = 17% = 50% = 33%	Grade 0=0 Grade 1=0 Grade 2=2 Grade3=10	= 0% = 0% = 17% = 83%	12
Papilloma	Grade 0=0 Grade 1=1 Grade 2=7 Grade 3=6	= 0% = 7% = 50% = 43%	Grade 0=0 Grade 1=0 Grade 2=0 Grade3=14	= 0% = 0% = 0% = 100%	14
Hyper/UDH	Grade 0=0 Grade 1=0 Grade 2=8 Grade 3=4	= 0% = 0% = 67% = 33%	Grade 0=0 Grade 1=0 Grade 2=1 Grade3=11	= 0% = 0% = 8% = 92%	12
ADH/LCIS/DCIS	Grade 0=0 Grade 1=0 Grade2=11 Grade 3=7	= 0% = 0% = 61% = 39%	Grade 0=0 Grade 1=0 Grade 2=0 Grade3=18	= 0% = 0% = 0% = 100%	18
Invasive cancer	Grade0=14 Grade 1=0 Grade 2=0 Grade 3=0	= 100% = 0% = 0% = 0%	Grade0=14 Grade 1=0 Grade 2=0 Grade 3=0	= 100% = 0% = 0% = 0%	14
Total	122		122		122

**Table 4:** Summary of the results of testing each individual group of lesions for any improvement in staining intensity using anti-CK 5 compared to anti-CK 5/6 using the McNemar test (one-sided)

Lesion	Anti CK5/6: Number of scores=3/total number of lesions	AntiCK5/6: % of total lesions with score=3	Anti-CK5: Number of scores=3/total number	Anti-CK5: % of total lesions with score=3	McNemar test *P-value* (one-sided)
Normal tissue	4/10	40%	10/10	100%	0.016
FCC/AM	4/16	25%	16/16	100%	0.002
SA	3/12	25%	12/12	100%	0.011
CCC	3/14	21.4%	14/14	100%	0.003
FA	4/12	33.3%	11/12	91.7%	0.035
Papilloma	6/14	42.9%	14/14	100%	0.020
Hyper/UDH	4/12	33.3%	11/12	91.7%	0.020
ADH/LCIS/DCIS	7/18	38.9%	18/18	100%	0.003

**Photographs of the stained slides of various breast lesions**

**Figure 1:**
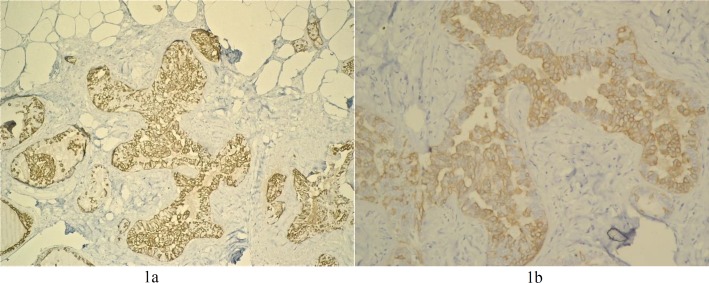
Usual ductal hyperplasia x20. Figure 1a (anti-CK5), Figure 1b (anti-CK56)

The examples of the slides photographed of a lesion stained with either anti-CK5/6 or with anti-CK5 are shown in Figure 1. The staining is discernably more intense in the anti-CK5 stained slide (figure 1a) compared to the anti-CK5/6 stained counterpart (figure 1b).

## Discussion

The specific role and use of anti-CK5/6 monoclonal antibody in breast disease diagnosis is well established. However, with the recent commercial availability of a new antibody, Leica microsystem anti-CK5 monoclonal antibody, there has been growing interest growing in its potential use as an alternative to the older anti-CK5/6. The uptake of the anti-CK5 antibody in global or UK routine clinical use is hitherto unknown, but it has been tried out on an *ad hoc* basis by the investigators. These *ad hoc* experiences have shown promising results with some evidence that the staining pattern (related to specificity) using anti-CK5 across a range of lesions and certainly for the lesions involved in the diagnostic conundrums described above, are the same as for anti-CK5/6 staining. Such initial *ad hoc* observations have also consistently suggested that most if not all lesions stained with anti-CK5, stain more intensely and therefore clearly (related to sensitivity) than those stained with anti-CK5/6. This would potentially make diagnosis more easily and with a lower risk of making false negative diagnoses for weakly staining yet potentially serious lesions. These initial observations formed the main basis of the rationale of this study to attempt to find evidence whether the anti-CK5 monoclonal antibody would indeed or otherwise stain a range of lesions with the same pattern and thus reach the same diagnosis, and also to investigate whether anti-CK5 stains a range of breast tissues/lesions with a greater intensity or otherwise compared to the staining with the anti-CK5/6 monoclonal antibody.

The results showed that the diagnoses resulting from using the standard diagnostic sequence of a combination of viewing the *H&E* stained slide, followed by either the anti-CK5 or the anti-CK5/6 stained slide is the same. It is also impressive that the results are 100% in concordance with the `gold standard` diagnoses list, which was based on reviewing the *H&E* slides with reference to the original pathology reports. The 100% diagnostic concordance seen using anti-CK5 and anti-CK5/6 in this study, attest to the fact that the sensitivity of anti-CK5 is at least as good as that of anti-CK5/6 over the range and sample population of lesions studied. These results thus reassuringly suggest that the risk of making a false negative diagnosis is no greater with using anti-CK5 antibody than with using the established anti-CK5/6 antibody. 

Comparing the distribution of the intensity scores of all lesions (except for invasive carcinoma which does not stain at all with either anti-CK5 or anti-CK5/6) diagnosed in both the anti-CK5/6 and the anti-CK5 stained populations, suggest strongly that staining with anti-CK5 results in much more strongly (clearer) staining lesions than anti-CK5/6 (Tables 2 and 3). There is also some evidence that this improvement in staining intensity is non-uniform between different lesions. Some of them show proportionally more improvement than others, but on the whole, all lesions show an improved staining intensity outcome with anti-CK5. Of notable mention, it seems there is a significant proportion of fibroadenomas and papillomas, which stain only weakly (score=1) with anti-CK5/6, and which all stained more intensely (score=2 or 3) when stained with anti-CK5. This has the implication that anti-CK5 may be more sensitive at picking up weakly staining lesions which may have the possible risk of being missed with anti-CK5/6 staining.

The statistical analysis of the data comparing the staining intensity scores for the individual lesions stained with either anti-CK5/6 or anti-CK5, using the McNemar test, provided quantitative evidence that all the different breast tissues/lesions stained with the anti-CK5, stained at a higher intensity (i.e. improved outcome) compared to the anti-CK5/6 (Table 4). Thus, in all the examined individual lesions (except arguably in the case of normal breast tissue), using the McNemar test, the derived *P-values* were all (practically) less than a critical value of set at α = 0.05. Thus, giving evidence at the 5% significance level that the breast lesions examined showed higher intensity when stained with the anti-CK5 compared to the anti-CK5/6. 

## Conclusion

Such evidence of comparable and indeed more favorable staining characteristics of anti-CK5 means that we may be justified in introducing anti-CK5 into everyday clinical practice, and maybe even replacing anti-CK5/6. Indeed, there are real cost benefits in using anti-CK5 compared to using anti-CK5/6. At the time this study was carried out, the Leica microsystems anti-CK5 antibody, NCL-CK5, had a list price of £219/ml compared to the Dako anti-CK5/6 antibody M7237 which had a list price of £308/ml. Replacing regular routine use of anti-CK5/6 with anti-CK5 should result in cost savings. In the current climate of government budget cutbacks, there is every pressure and incentive to make savings in every department, whenever and wherever possible. The authors propose that histopathology services should consider replacing the use of anti-CK5/6 with anti-CK5, as it has comparable if not better diagnostic characteristics and should also result in budget savings.
